# National trends in dual de-escalation of axillary surgery and adjuvant radiation in early-stage HR+/HER2- breast cancer

**DOI:** 10.1007/s10549-026-08106-0

**Published:** 2026-10-18

**Authors:** Dylan S. Brokaw, Leonardo Kozian, Cecilia Chang, Rebecca M. Shulman, Richard J. Bleicher, Alycia So, Allison A. Aggon, Austin D. Williams

**Affiliations:** 1 Division of Breast Surgery, Department of Surgery, Fox Chase Cancer Center, 333 Cottman Ave, c308, Philadelphia, PA 19111 USA; 2 Department of Surgery, Indiana University School of Medicine, Indianapolis, IN USA; 3 Research Institute, Endeavor Health, Evanston, IL USA; 4 Department of Radiation Oncology, Fox Chase Cancer Center, Philadelphia, PA USA

**Keywords:** Breast cancer, De-escalation, SLNB omission, APBI, Axillary surgery, Practice patterns

## Abstract

**Purpose:**

De-escalation has become standard across breast cancer management. Many early-stage HR+/HER2-negative breast cancers are eligible for both SLNB omission and accelerated partial breast irradiation (APBI). However, the oncologic safety of dual de-escalation has not been evaluated except in patients ≥ 70 years. We sought to characterize national practice patterns and factors associated with adoption of this dual de-escalation approach.

**Methods:**

Women aged 50–69 years with clinical T1–T2N0 breast cancer who underwent upfront breast-conserving surgery between (2012 − 2022) were identified from the National Cancer Database. Patients were categorized by axillary surgery and by adjuvant radiation modality (WBI, APBI, or none). We assessed trends and factors associated with de-escalation.

**Results:**

316,324 patients met inclusion criteria, of whom 98% underwent SLNB. Among patients who underwent SLNB, 83% received WBI, 11% received APBI, and 6% received no radiation. In contrast, among patients in whom SLNB was omitted, 63% received WBI, 8% received APBI, and 30% received no radiation. SLNB omission remained low throughout the study period at < 3%, while APBI use increased from 8% to 16.5%. Dual de-escalation was rarely performed throughout the study period remaining < 0.30%. Shared predictors of de-escalation included cT1 tumors, ductal histology, low grade, and treatment at academic/research programs.

**Conclusion:**

These findings highlight the need for prospective studies to define the safety, outcomes, and patient selection criteria for combined de-escalation strategies. Until these data are available, patient-centered discussions are essential to determine the most appropriate strategy for patients with low-risk disease.

## Introduction

De-escalation of breast cancer care has become a central paradigm in the management of early-stage disease. This shift has been driven by improved tumor biology stratification, advances in systemic therapy, and an increasing emphasis on minimizing treatment-related morbidity without compromising oncologic outcomes. Across surgical, radiation, and medical oncology, randomized trials have demonstrated that carefully selected patients may safely receive less intensive therapy while maintaining excellent local control and survival.

Axillary management has been a major focus of surgical de-escalation. Sentinel lymphadenectomy (SLNB), while less morbid than axillary lymph node dissection (ALND), remains associated with risks including lymphedema, pain, and reduced shoulder mobility [1]. The SOUND [2], INSEMA [3], and BOOG 2013-08 [4] trials provided evidence that omission of SLNB is safe among patients ≥ 50 with early-stage hormone receptor (HR)+/HER2- breast cancer undergoing breast conserving surgery (BCS) with negative preoperative axillary ultrasound. These findings have accelerated interests in selective omission of axillary surgery in low-risk patients and have begun to influence clinical practice [5].

Concurrently, there has been a shift toward shorter, more targeted radiation regimens for BCS patients. Accelerated partial breast irradiation (APBI) has emerged as an alternative to whole breast irradiation (WBI) for appropriately selected patients, offering convenience, reduced exposure of normal tissues, less morbidity and comparable oncologic outcomes in randomized trials [6, 7]. Current guidelines support APBI for patients with favorable clinicopathologic features [8], many of whom are also eligible for SLNB omission.

Despite many patients meeting criteria for both surgical and radiation de-escalation, their combined use has not been well studied. Nearly all patients in the randomized trials investigating SLNB omission underwent WBI, since there may be some treatment effect of the low axilla when WBI is performed [9]. The oncologic safety of dual de-escalation (SLNB omission and limiting radiation) has only been explored in limited cohorts, primarily among patients ≥ 70 [9]. As a result, clinicians face uncertainty regarding whether these de-escalation strategies can be safely adopted together in routine practice.

Understanding real-world practice patterns is essential to inform future trial design, guideline development, and shared decision-making. Therefore, we sought to characterize national trends in surgical de-escalation, radiation de-escalation, and their combined use among women aged 50–69 with early-stage HR+/HER2- breast cancers treated with breast conserving therapy.

## Methods

We performed a retrospective cohort study using the National Cancer Database (NCDB), a hospital-based registry administered by the American College of Surgeons that captures patient-level data from Commission on Cancer–accredited programs and represents approximately 70% of newly diagnosed cancer cases in the United States [10, 11]. As the NCDB contains de-identified data, this study was exempt from institutional review board oversight. From the NCDB breast cancer Participant User File, we identified women aged 50–69 diagnosed between 2012 and 2022 with clinical T1-T2 N0 HR+/HER2-negative invasive breast cancer treated with upfront BCS. Hormone receptor positivity was defined as estrogen receptor and/or progesterone receptor positivity, and HER2- negative status was defined according to NCDB coding standards. Patients were excluded if they received neoadjuvant systemic therapy, underwent mastectomy, had clinically node-positive disease, or had missing data.

Axillary surgery was defined as coded by the Participant User File and categorized as none (SLNB omission), SLNB alone, or SLNB with completion ALND. Radiation modality was categorized as APBI, WBI (conventional or moderately hypofractionated), or no radiation, using the NCDB fields for Phase I radiation treatment volume, total dose, and number of fractions. Only external-beam radiation was included. Patients receiving brachytherapy or intraoperative radiation, ultrahypofractionated WBI, or regimens with unknown or unclassifiable dose or fractionation were excluded. APBI was utilized as an indication for intentional radiation de-escalation, whereas nonreceipt of radiation was noted to be separate as intent of nonreceipt was unable to be determined in this analysis. Accordingly, dual de-escalation was defined as the combination of SLNB omission and receipt of APBI. Patients undergoing SLNB omission without receipt of any radiation were analyzed as a distinct category and were not classified as dual de-escalation. Patient-level covariates assessed included age, race, ethnicity, insurance status, median household income, education, urban versus rural residence, Charlson-Deyo comorbidity score, and facility type. Tumor-level covariates included clinical tumor stage, histology, and grade.

### Statistical analysis

Descriptive statistics were used to summarize baseline characteristics and treatment patterns. Comparisons between axillary surgery types and radiation modalities were performed using Chi-squared tests and t-tests or ANOVA, as appropriate Temporal trends in axillary surgery and radiotherapy were evaluated across the study period. Multivariable logistic regression models were constructed to identify factors independently associated with surgical de-escalation, radiation de-escalation, and dual de-escalation. The comprehensive list of patient, socioeconomic, and tumor-level covariates included in each model is detailed in Tables 3, 4 and 5. Results were reported as odds ratios (ORs) with 95% confidence intervals (CI). Statistical significance was defined as *p* < 0.05. All analyses were performed using SAS 9.4 (SAS Institute Inc., Cary, NC).

## Results

We identified 316,324 female patients with clinical T1-T2 N0 HR+/HER2-negative invasive breast cancer who underwent upfront BCS (Table 1). The mean age was 60.1 ± 5.6 years, most patients had clinical T1 tumors (86%), low (35%) or intermediate (48.5%) grade disease, and were treated at comprehensive community or academic centers. Most patients had a Charlson-Deyo comorbidity score of 0 (83.5%).

**Table 1:** Clinicopathologic features and treatment strategies for patients 50–69 with cT1-2 N0 HR+HER2- breast cancer undergoing breast conserving surgery stratified by performance of sentinel lymphadenectomy and breast radiation approach

		SLNB				No SLNB				
	Overall	WBI	APBI	No radiation	p1	WBI	APBI	No radiation	p2	p3
**n**	316,324	256,418	33,970	19,567		3,985	496	1,888		
**Age (years)**,** mean ± SD**	60.8 ± 5.6	60.6 ± 5.6	61.3 ± 5.4	62.3 ± 5.6	< 0.001	61.9 ± 5.6	62.6 ± 5.4	63.0 ± 5.5	< 0.001	< 0.001
**Race/Ethnicity**					< 0.001				0.04	< 0.001
White	269,023 (85.1)	218,183 (85.1)	29,381 (86.5)	16,151 (82.5)		3308 (83.0)	433 (87.3)	1567 (83.0)		
Black	27,830 (8.8)	22,377 (8.7)	2646 (7.8)	2121 (10.8)		424 (10.6)	41 (8.3)	221 (11.7)		
Asian	13,366 (4.2)	11,004 (4.3)	1308 (3.9)	828 (4.2)		155 (3.9)	*	54 (2.9)		
Other/Unknown	6105 (1.9)	4854 (1.9)	635 (1.9)	467 (2.4)		98 (2.5)	*	46 (2.4)		
**Charlson/Deyo Score**					< 0.001				0.0005	< 0.001
0	263,868 (83.4)	214,547 (83.7)	28,568 (84.1)	15,810 (80.8)		3149 (79.0)	393 (79.2)	1401 (74.2)		
1	39,522 (12.5)	32,010 (12.5)	4090 (12.0)	2553 (13.1)		528 (13.3)	57 (11.5)	284 (15.0)		
2	8696 (2.8)	6733 (2.6)	897 (2.6)	759 (3.9)		175 (4.4)	27 (5.4)	105 (5.6)		
≥ 3	4238 (1.3)	3128 (1.2)	415 (1.2)	445 (2.3)		133 (3.3)	19 (3.8)	98 (5.2)		
**Insurance Status**					< 0.001				< 0.001	< 0.001
Not Insured	3667 (1.2)	2940 (1.2)	300 (0.9)	325 (1.7)		63 (1.6)	*	33 (1.8)		
Private Insurance	190,381 (60.2)	158,163 (61.7)	20,410 (60.1)	8966 (45.8)		1928 (48.4)	236 (47.6)	678 (35.9)		
Medicaid	18,612 (5.9)	15,088 (5.9)	1582 (4.7)	1474 (7.5)		287 (7.2)	27 (5.4)	154 (8.2)		
Medicare	96,709 (30.6)	74,579 (29.1)	10,954 (32.3)	8383 (42.8)		1585 (39.8)	215 (43.4)	993 (52.6)		
Other Government	4113 (1.3)	3370 (1.3)	444 (1.3)	222 (1.1)		62 (1.6)	*	10 (0.5)		
Unknown	2842 (0.9)	2278 (0.9)	280 (0.8)	197 (1.0)		60 (1.5)	*	20 (1.1)		
**No High School Degree**					< 0.001				0.45	< 0.001
>=15.3%	42,346 (13.4)	33,818 (13.2)	4265 (12.6)	3229 (16.5)		648 (16.3)	89 (17.9)	297 (15.7)		
9.1% − 15.2%	68,645 (21.7)	55,113 (21.5)	7559 (22.3)	4499 (23.0)		936 (23.5)	103 (20.8)	435 (23.0)		
5.0% − 9.0%	82,399 (26.1)	66,971 (26.1)	8918 (26.3)	4904 (25.1)		999 (25.1)	141 (28.4)	466 (24.7)		
< 5.0%	73,341 (23.2)	60,243 (23.5)	7785 (22.9)	3932 (20.1)		853 (21.4)	102 (20.6)	426 (22.6)		
Unknown	49,593 (15.7)	40,273 (15.7)	5443 (16.0)	3003 (15.4)		549 (13.8)	61 (12.3)	264 (14.0)		
**Median Income**					< 0.001				0.6	< 0.001
< $46,277	32,545 (10.3)	25,984 (10.1)	3319 (9.8)	2433 (12.4)		502 (12.6)	65 (13.1)	242 (12.8)		
$46,277 - $57,856	50,689 (16.0)	40,781 (15.9)	5454 (16.1)	3375 (17.3)		658 (16.5)	96 (19.4)	325 (17.2)		
$57,857 - $74,062	63,806 (20.2)	51,714 (20.2)	6721 (19.8)	4114 (21.0)		774 (19.4)	103 (20.8)	380 (20.1)		
>=$74,063	119,103 (37.7)	97,216 (37.9)	12,955 (38.1)	6600 (33.7)		1490 (37.4)	171 (34.5)	671 (35.5)		
Unknown	50,181 (15.9)	40,723 (15.9)	5521 (16.3)	3045 (15.6)		561 (14.1)	61 (12.3)	270 (14.3)		
**Institution Type**					< 0.001				< 0.001	< 0.001
Community Cancer Center	22,730 (7.2)	18,627 (7.3)	2061 (6.1)	1526 (7.8)		331 (8.3)	21 (4.2)	164 (8.7)		
Comprehensive Community Cancer Program	129,627 (41.0)	106,148 (41.4)	12,925 (38.1)	7960 (40.7)		1698 (42.6)	197 (39.7)	699 (37.0)		
Academic/Research Program	94,498 (29.9)	75,179 (29.3)	11,695 (34.4)	5532 (28.3)		1250 (31.4)	174 (35.1)	668 (35.4)		
Integrated Network Cancer Program	69,469 (22.0)	56,464 (22.0)	7289 (21.5)	4549 (23.3)		706 (17.7)	104 (21.0)	357 (18.9)		
**Clinical Tumor Stage**					< 0.001				< 0.001	0.41
cT1	270,640 (85.6)	217,736 (84.9)	30,991 (91.2)	16,441 (84.0)		3455 (86.7)	456 (91.9)	1561 (82.7)		
cT2	45,684 (14.4)	38,682 (15.1)	2979 (8.8)	3126 (16.0)		530 (13.3)	40 (8.1)	327 (17.3)		
**Tumor Histology**					< 0.001				0.04	< 0.001
Ductal	281,706 (89.1)	227,232 (88.6)	31,129 (91.6)	17,605 (90.0)		3588 (90.0)	467 (94.2)	1685 (89.3)		
Lobular	33,239 (10.5)	28,048 (10.9)	2737 (8.1)	1877 (9.6)		368 (9.2)	27 (5.4)	182 (9.6)		
Mixed/Other	1165 (0.4)	959 (0.4)	95 (0.3)	70 (0.4)		*	*	*		
Unknown	214 (0.1)	179 (0.1)	9 (0.0)	15 (0.1)		*	*	*		
**Tumor Grade**					< 0.001				< 0.001	< 0.001
Low	110,855 (35.0)	87,365 (34.1)	13,665 (40.2)	7241 (37.0)		1552 (39.0)	220 (44.4)	812 (43.0)		
Intermediate	153,261 (48.5)	125,661 (49.0)	16,212 (47.7)	8808 (45.0)		1702 (42.7)	182 (36.7)	696 (36.9)		
High	39,257 (12.4)	33,212 (13.0)	2919 (8.6)	2510 (12.8)		400 (10.0)	36 (7.3)	180 (9.5)		
Unknown	12,951 (4.1)	10,180 (4.0)	1174 (3.5)	1008 (5.2)		331 (8.3)	58 (11.7)	200 (10.6)		
**Sentinel lymph nodes removed**,** mean ± SD**	2.5 ± 1.7	2.50 ± 1.7	2.4 ± 1.6	2.5 ± 1.7	< 0.001					
**Sentinel lymph nodes positive**,** mean ± SD**	0.2 ± 0.5	0.2 ± 0.5	0.1 ± 0.4	0.2 ± 0.6	< 0.001					
SLNs positive	19,850 (12.4)	17,284 (13.6)	1382 (6.1)	1184 (10.8)						
SLNs negative	140,721 (87.6)	109,750 (86.4)	21,155 (93.9)	9816 (89.2)						
**Axillary lymph node dissection**										
No	287,848 (91.0)	232,171 (90.5)	31,639 (93.1)	17,669 (90.3)	< 0.001					
Yes	28,476 (9.0)	24,247 (9.5)	2331 (6.9)	1898 (9.7)						
Additional lymph nodes removed	3.50 ± 5.32	3.63 ± 5.41	2.52 ± 4.17	3.62 ± 5.79	< 0.001					
Additional lymph nodes positive	0.41 ± 1.79	0.42 ± 1.74	0.23 ± 1.28	0.64 ± 2.70	< 0.001					

* Results suppressed: The National Cancer Database does not permit aggregate results for cell sizes < 10

p1 and p2 represent comparisons of radiation groups within the groups of patients for whom SLNB was and was not performed, respectively. p3 represents comparison of SLNB and no SLNB

Note: Table cells show frequency (column percent) or mean ± standard deviation (SD)

Abbreviations: SLNB: sentinel lymphadenectomy; WBI: whole breast irradiation; APBI: accelerated partial breast irradiation; SLN: sentinel lymph node

**Fig. 1:**
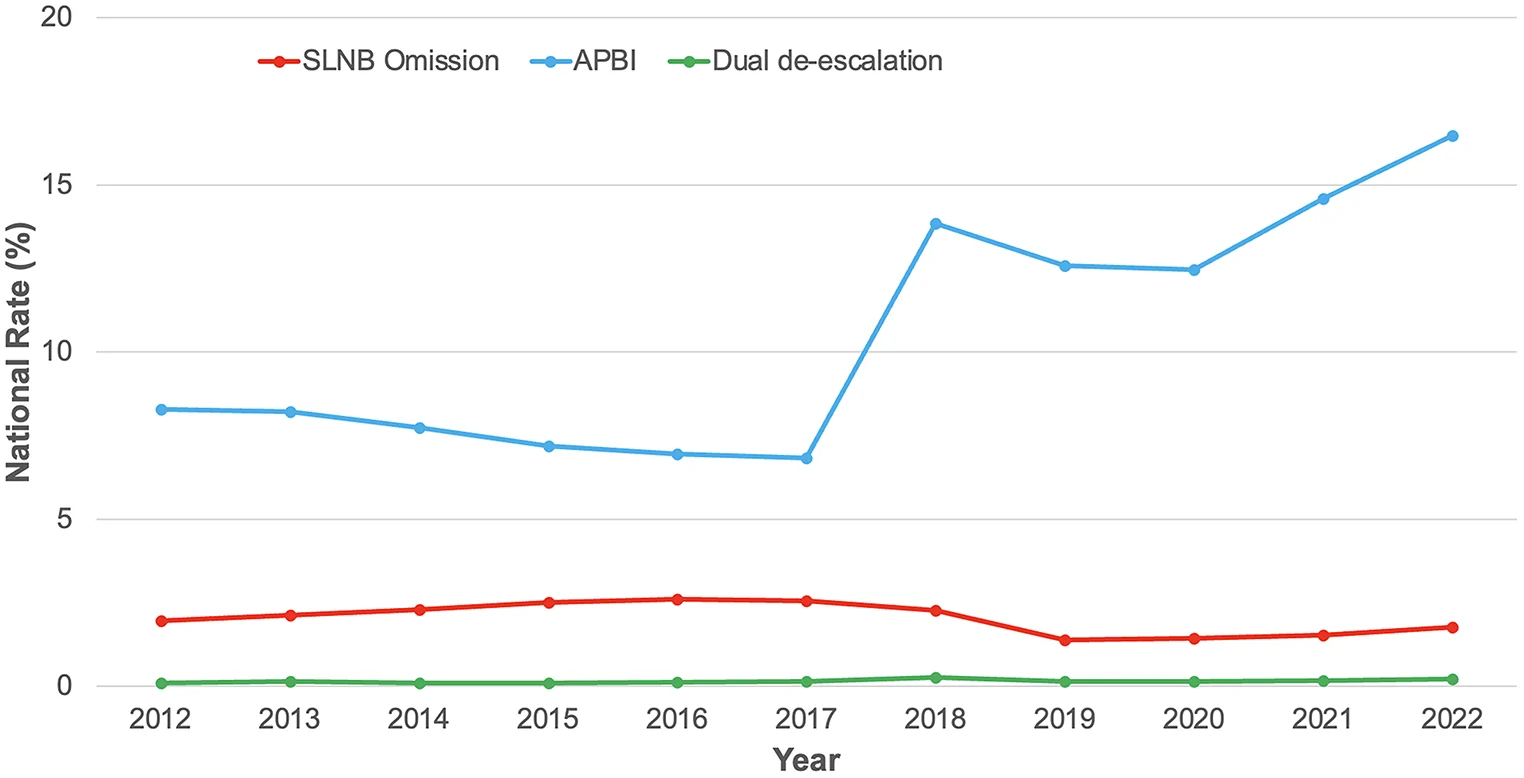
Rates of omission of sentinel lymphadenectomy (SLNB), use of accelerated partial breast irradiation (APBI) and dual de-escalation (SLNB+APBI) among patients 50–69 with cT1-2 N0 HR+HER2- breast cancer undergoing breast conserving surgery (2012–2022)

SLNB was omitted in only 2% of patients overall; 89% underwent SLNB alone and 9% had SLNB+ALND. Patients in whom SLNB was omitted were older than those undergoing SLNB (62.2 ± 5.6 v. 60.8 ± 5.6, *p* < 0.001), and were more likely to have low-grade tumors (40.6% v. 34.9%, *p* < 0.001), but had similar rates of cT2 cancers (14.1% v. 14.4%, *p* = 0.41). Over the study period, ALND use declined steadily from 16.4% in 2012 to 4.2% in 2022, while SLNB correspondingly increased from 81.6% to 94%. SLNB omission remained uncommon throughout the study period, increasing from 2% in 2012 to a peak of 2.6% in 2016, then declining to a nadir of 1.4% in 2019 and partially recovering to 1.8% by 2022 (Fig. 1). Patients undergoing SLNB had a mean of 2.5 ± 1.7 sentinel nodes (SLNs) removed, and 12.4% had a positive sentinel node. Among these patients, the mean number of positive nodes was approximately 1.3. For those who went on to complete ALND, a mean of 3.5 ± 5.3 additional nodes were removed (Table 1).

Overall, radiation was omitted in 7% of patients, while 82% underwent WBI and 11% underwent APBI. APBI utilization remained below 8.5% through 2017, before nearly doubling in 2018 from 6.8% to 13.9%, and continuing to rise to 16.5% by 2022 (Fig. 1). In patients where SLNB was performed, APBI compared to WBI was performed more in academic/research programs (34.4% vs. 29.3%, *p* < 0.001) and cT1 cancers (91.2% v 84.9%, *p* < 0.001). APBI use was less common among patients with lobular cancers (8.1% v 10.9%, *p* < 0.001) and high grade tumors (8.6% v 13.0%, *p* < 0.001) compared with patients receiving WBI. Similar patterns were observed among patients undergoing dual de-escalation. Compared with patients who underwent SLNB omission followed by WBI, those receiving dual de-escalation were more frequently treated at academic/research programs (35.1% v 31.4%, *p* < 0.001) and more often had cT1 disease (91.9% v 86.7%, *p* < 0.001). Similarly, dual de-escalation was less common amongst patients with lobular cancers (5.4% v 9.2%, *p* < 0.05) and high-grade tumors (7.3%, v 10%, *p* < 0.001) (Table 1). Dual de-escalation remained rare throughout the study period, occurring in fewer than 0.30% of patients in any given year. A modest increase was observed beginning in 2018 (0.26%), before settling at 0.23% by 2022 (Fig. 1).

Pathologic tumor size, nodal stage, and axillary surgery performed were also associated with radiation received. Most patients across all treatment groups had pT1 cancers (overall: 80.6%). The APBI group had the highest proportion of pT1 tumors (87.9%), compared with the WBI (80.0%) and no radiation (76.7%) groups. pT2 disease was most prevalent in the no radiation group (18.21%) relative to the WBI (17.3%) and APBI (9.8%) groups. Differences in pathologic tumor stage across treatment groups were statistically significant (*p* < 0.001). Pathologic nodal stage also differed significantly across groups (*p* < 0.001). Node-negative disease (pN0) was most common in the APBI group (92.9%), compared to WBI (85.2%) and no radiation (81.0%) groups. pN1 disease was more frequent in the WBI (11.4%) and no radiation (8.8%) groups than in the APBI group (4.4%) (Table 2).

**Table 2:** Clinical approach and pathologic stage of patients 50–69 with cT1-2N0 breast cancer

	Overall	WBI	APBI	No Radiation	*p*
**n**	329,741	271,614	35,577	22,550	
**Pathologic Tumor Stage**					< 0.001
pT0	695 (0.2)	558 (0.2)	55 (0.2)	82 (0.4)	
pTis	372 (0.1)	280 (0.1)	50 (0.1)	42 (0.2)	
pT1	265,779 (80.6)	217,228 (80.0)	31,258 (87.9)	17,293 (76.7)	
pT2	54,562 (16.6)	46,973 (17.3)	3483 (9.8)	4106 (18.2)	
pT3	1420 (0.4)	1195 (0.4)	55 (0.2)	170 (0.8)	
pT4	95 (0.0)	76 (0.0)	*	15 (0.1)	
pTX	841 (0.3)	591 (0.2)	*	149 (0.7)	
Unknown	5977 (1.8)	4713 (1.7)	571 (1.6)	693 (3.1)	
**Pathologic Nodal Stage**					< 0.001
pN0	282,620 (85.7)	231,296 (85.2)	33,061 (92.9)	18,263 (81.0)	
pN1	34,508 (10.5)	30,957 (11.4)	1564 (4.4)	1987 (8.8)	
pN2	2132 (0.7)	1849 (0.7)	64 (0.2)	219 (1.0)	
pN3	612 (0.2)	503 (0.2)	17 (0.1)	92 (0.4)	
pNX	3637 (1.1)	2143 (0.8)	313 (0.9)	1181 (5.2)	
Unknown	6232 (1.9)	4866 (1.8)	558 (1.6)	808 (3.6)	
**Axillary Surgery**					< 0.001
SLNB Omission	6369 (2.0)	3985 (1.5)	496 (1.4)	1888 (8.8)	
SLNB	281,479 (89.0)	232,171 (89.2)	31,639 (91.8)	17,669 (82.4)	
SLNB + ALND	28,476 (9.0)	24,247 (9.3)	2331 (6.8)	1898 (8.9)	

* Results suppressed: The National Cancer Database does not permit aggregate results for cell sizes < 10

Note: Table cells show frequency (column percent)

### Predictors of de-escalation

We performed univariate and multivariable analyses to assess factors associated with SLNB omission, APBI receipt, and dual de-escalation. Older age and higher comorbidity burden were each associated with greater odds of SLNB omission (Table 3). Treatment at academic/research programs was associated with nearly twice the odds of SLNB omission compared to community cancer centers (OR 1.85, *p* < 0.001). Higher tumor grade, clinical tumor stage, and lobular histology were each associated with lower odds of SLNB omission (all *p* < 0.001). Notably, receipt of either WBI (OR 0.296) or APBI (OR 0.32) was associated with markedly lower odds of SLNB omission compared to no radiation (both *p* < 0.001).

With respect to APBI receipt rather than WBI, Black and Asian patients were significantly less likely to receive APBI compared to White patients (OR 0.87 and 0.88, respectively; both *p* < 0.001, Table 4), while Medicare and private insurance were associated with higher odds of APBI receipt compared with uninsured status. Treatment at academic programs was associated with higher odds of APBI use compared with community cancer centers (OR 1.43, *p* < 0.001). Clinical T2 tumors (OR 0.62), higher tumor grade (High: OR 0.689, *p* < 0.0001; Intermediate: OR 0.908, *p* < 0.0001), and lobular histology (OR 0.712, *p* < 0.0001) were each associated with lower odds of APBI (all *p* < 0.001. Performance of SLNB was the strongest predictor of APBI use (OR 1.89, *p* < 0.001).

**Table 3:** Univariate and multivariable analysis of factors predictive of SLNB omission

	UNIVARIATE			MULTIVARIABLE		
	OR	95% CI	*p*	OR	95% CI	*p*
**Age (years)**	1.17	1.17–1.17	< 0.001	1.14	1.14–1.14	< 0.001
**Race/Ethnicity**						
White (ref)	-	-	-	-	-	-
Black	0.88	0.85–0.92	< 0.001	1.08	1.03–1.14	0.002
Asian	0.80	0.76–0.85	< 0.001	1.05	0.98–1.13	0.14
Other/Unknown	0.84	0.78–0.91	< 0.001	1.02	0.92–1.13	0.66
**Charlson/Deyo Score**						
0 (ref)	-	-	-	-	-	-
1	1.30	1.26–1.33	< 0.001	1.10	1.06–1.14	< 0.001
2	2.07	1.99–2.16	< 0.001	1.42	1.34–1.50	< 0.001
≥ 3	2.94	2.80–3.08	< 0.001	1.77	1.66–1.89	< 0.001
**Insurance Status**						
Not Insured (ref)	-	-	-	-	-	-
Private Insurance	0.59	0.51–0.68	< 0.001	0.67	0.56–0.80	< 0.001
Medicaid	0.97	0.83–1.13	0.7100	1.04	0.86–1.26	0.69
Medicare	3.33	2.88–3.83	< 0.001	0.73	0.61–0.87	0.001
Other Government	1.11	0.92–1.34	0.27	0.72	0.57–0.92	0.007
**No High School Degree**						
>=15.3% (ref)	-	-	-	-	-	-
9.1% − 15.2%	1.03	1.00-1.07	0.10	0.97	0.93–1.02	0.22
5.0% − 9.0%	1.06	1.02–1.09	0.001	0.93	0.89–0.97	0.001
< 5.0%	1.15	1.12–1.19	< 0.001	0.95	0.91-1.00	0.06
**Median Income**						
< $46,277 (ref)	-	-	-	-	-	-
$46,277 - $57,856	0.97	0.93-1.00	0.08	0.99	0.95–1.04	0.82
$57,857 - $74,062	1.02	0.98–1.06	0.39	1.11	1.06–1.17	< 0.001
>=$74,063	1.11	1.07–1.15	< 0.001	1.33	1.27–1.40	< 0.001
**Institution Type**						
Community Cancer Center (ref)	-	-	-	-	-	-
Comprehensive Community Cancer Program	1.10	1.06–1.14	< 0.001	1.19	1.13–1.25	< 0.001
Academic/Research Program	1.35	1.30–1.41	< 0.001	1.85	1.76–1.95	< 0.001
Integrated Network Cancer Program	1.16	1.11–1.21	< 0.001	1.37	1.30–1.45	< 0.001
**Clinical Tumor Stage**						
cT1 (ref)	-	-	-	-	-	-
cT2	1.00	0.97–1.02	0.76	0.88	0.85–0.91	< 0.001
**Tumor Histology**						
Ductal (ref)	-	-	-	-	-	-
Lobular	0.98	0.95–1.01	0.11	0.88	0.85–0.92	< 0.001
Mixed/Other	1.12	0.97–1.30	0.13	1.20	0.99–1.47	0.07
**Tumor Grade**						
Low (ref)	-	-	-	-	-	-
Intermediate	0.86	0.84–0.87	< 0.001	0.82	0.80–0.84	< 0.001
High						< 0.001
**Radiation Therapy**						
No Radiation (ref)	-	-	-	-	-	-
WBI	0.10	0.10–0.11	< 0.001	0.30	0.29–0.30	< 0.001
APBI	0.13	0.13–0.14	< 0.001	0.32	0.30–0.33	< 0.001

**Table 4:** Univariate and multivariable analysis of factors predictive of APBI

	UNIVARIATE			MULTIVARIABLE		
	OR	95% CI	*p*	OR	95% CI	*p*
**Age (years)**	0.99	0.99–0.99	< 0.001	0.99	0.99–0.99	< 0.001
**Race/Ethnicity**						
White (ref)	-	-	-	-	-	-
Black	0.87	0.84–0.91	< 0.001	0.87	0.84–0.91	< 0.001
Asian	0.92	0.88–0.97	0.001	0.88	0.83–0.93	< 0.001
Other/Unknown	0.97	0.91–1.04	0.44	0.96	0.89–1.04	0.3300
**Charlson/Deyo Score**						
0 (ref)	-	-	-	-	-	-
1	0.92	0.90–0.95	< 0.001	0.96	0.93–0.99	0.004
2	0.88	0.84–0.93	< 0.001	0.96	0.90–1.02	0.1500
≥ 3	0.79	0.74–0.85	< 0.001	0.90	0.83–0.97	0.01
**Insurance Status**						
Not Insured (ref)	-	-	-	-	-	-
Private Insurance	1.33	1.19–1.48	< 0.001	1.28	1.13–1.45	< 0.001
Medicaid	1.05	0.93–1.18	0.44	1.04	0.91–1.19	0.5700
Medicare	1.20	1.07–1.34	0.002	1.39	1.23–1.58	< 0.001
Other Government	1.28	1.11–1.48	< 0.001	1.34	1.14–1.57	< 0.001
**No High School Degree**						
>=15.3% (ref)	-	-	-	-	-	-
9.1% − 15.2%	1.07	1.04–1.11	< 0.001	1.05	1.01–1.08	0.0100
5.0% − 9.0%	1.06	1.02–1.09	< 0.001	1.00	0.96–1.03	0.8800
< 5.0%	1.06	1.03–1.10	< 0.001	0.96	0.92-1.00	0.0500
**Median Income**						
< $46,277 (ref)	-	-	-	-	-	-
$46,277 - $57,856	1.06	1.02–1.10	0.002	1.04	1.00-1.08	0.0700
$57,857 - $74,062	1.04	1.01–1.08	0.02	1.02	0.98–1.06	0.4400
>=$74,063	1.10	1.06–1.13	< 0.001	1.07	1.02–1.11	0.0020
**Institution Type**						
Community Cancer Center (ref)	-	-	-	-	-	-
Comprehensive Community Cancer Program	1.15	1.10–1.19	< 0.001	1.11	1.06–1.16	< 0.001
Academic/Research Program	1.43	1.38–1.49	< 0.001	1.43	1.37–1.50	< 0.001
Integrated Network Cancer Program	1.19	1.14–1.24	< 0.001	1.13	1.08–1.18	< 0.001
**Clinical Tumor Stage**						
cT1 (ref)	-	-	-	-	-	-
cT2	0.56	0.54–0.58	< 0.001	0.62	0.60–0.64	< 0.001
**Tumor Histology**						
Ductal (ref)	-	-	-	-	-	-
Lobular	0.70	0.68–0.72	< 0.001	0.71	0.69–0.74	< 0.001
Mixed/Other	0.71	0.60–0.84	< 0.001	0.86	0.71–1.05	0.1300
**Tumor Grade**						
Low (ref)	-	-	-	-	-	-
Intermediate	0.87	0.85–0.89	< 0.001	0.91	0.89–0.93	< 0.001
High	0.64	0.62–0.66	< 0.001	0.69	0.66–0.72	< 0.001
**Axillary Surgery**						
No Axillary Surgery (ref)	-	-	-	-	-	-
SLNB	1.95	1.88–2.03	< 0.001	1.89	1.80–1.98	< 0.001
ALND	1.41	1.34–1.49	< 0.001	1.41	1.33–1.50	< 0.001

Factors associated with dual de-escalation followed a similar pattern (Table 5). Older age was associated with slightly greater odds of dual de-escalation (OR 1.09, *p* < 0.001). Treatment at academic/research programs was associated with more than twice the odds of dual de-escalation compared with community cancer centers (OR 2.24, *p* < 0.001). Clinical T2 disease (OR 0.47, *p* < 0.001), higher tumor grade (intermediate: OR 0.80; high: 0.61, both *p* < 0.0001), and lobular histology (OR 0.83, *p* = 0.009) were each independently associated with lower odds of dual de-escalation.

**Table 5:** Univariate and multivariable analysis of factors predictive of dual de-escalation

	UNIVARIATE			MULTIVARIABLE		
	OR	95% CI	*p*	OR	95% CI	*p*
**Age (years)**	1.09	1.09–1.10	< 0.001	1.09	1.08–1.09	< 0.001
**Race/Ethnicity**						
White (ref)	-	-	-	-	-	-
Black	0.80	0.68–0.93	0.004	0.94	0.79–1.13	0.52
Asian	0.91	0.74–1.12	0.38	1.10	0.86–1.40	0.44
Other/Unknown	0.93	0.69–1.25	0.63	1.04	0.73–1.47	0.84
**Charlson/Deyo Score**						
0 (ref)	-	-	-	-	-	-
1	1.07	0.96–1.19	0.25	0.90	0.79–1.03	0.12
2	1.64	1.39–1.94	< 0.001	1.20	0.99–1.46	0.07
≥ 3	1.87	1.52–2.29	< 0.001	1.30	1.02–1.64	0.03
**Insurance Status**						
Not Insured (ref)	-	-	-	-	-	-
Private Insurance	0.89	0.47–1.69	0.72	0.89	0.43–1.84	0.76
Medicaid	1.18	0.59–2.35	0.63	1.30	0.60–2.82	0.50
Medicare	3.55	1.87–6.72	< 0.001	1.39	0.68–2.86	0.37
Other Government	1.61	0.74–3.53	0.23	1.46	0.61–3.46	0.39
**No High School Degree**						
>=15.3% (ref)	-	-	-	-	-	-
9.1% − 15.2%	0.95	0.83–1.09	0.46	0.85	0.73–0.99	0.04
5.0% − 9.0%	1.08	0.95–1.24	0.23	0.91	0.78–1.06	0.24
< 5.0%	1.19	1.04–1.36	0.01	0.95	0.81–1.13	0.57
**Median Income**						
< $46,277 (ref)	-	-	-	-	-	-
$46,277 - $57,856	1.11	0.95–1.31	0.19	1.14	0.95–1.35	0.1600
$57,857 - $74,062	1.21	1.04–1.42	0.01	1.23	1.03–1.47	0.0200
>=$74,063	1.25	1.08–1.44	0.003	1.28	1.07–1.53	0.0070
**Institution Type**						
Community Cancer Center (ref)	-	-	-	-	-	-
Comprehensive Community Cancer Program	1.54	1.27–1.87	< 0.001	1.39	1.13–1.71	0.0020
Academic/Research Program	2.11	1.74–2.55	< 0.001	2.24	1.82–2.75	< 0.001
Integrated Network Cancer Program	1.65	1.36–2.01	< 0.001	1.69	1.36–2.10	< 0.001
**Clinical Tumor Stage**						
cT1 (ref)	-	-	-	-	-	-
cT2	0.49	0.43–0.57	< 0.001	0.47	0.40–0.55	< 0.001
**Tumor Histology**						
Ductal (ref)	-	-	-	-	-	-
Lobular	0.85	0.74–0.96	0.01	0.83	0.71–0.95	0.009
Mixed/Other	0.74	0.36–1.51	0.41	0.82	0.36–1.90	0.6400
**Tumor Grade**						
Low (ref)	-	-	-	-	-	-
Intermediate	0.80	0.74–0.87	< 0.001	0.80	0.73–0.87	< 0.001
High	0.53	0.46–0.62	< 0.001	0.61	0.51–0.72	< 0.001

## Discussion

In this national analysis of women aged 50–69 with early-stage HR+/HER2- breast cancer, we observed increasing use of APBI, whereas SLNB omission remained uncommon. Dual de-escalation was rare, but a minor emergence was observed across the study period peaking slightly in 2018 before plateauing, suggesting that a subset of clinicians began implementing combined de-escalation strategies prior to formal prospective validation or guideline endorsement. Notably, our study period preceded the publication of practice-changing randomized trials supporting omission of SLNB, further highlighting early adoption of de-escalation strategies in carefully selected patients.

Importantly, de-escalation was not applied broadly, but rather concentrated among patients with favorable clinicopathologic features. We found that larger tumor size and higher grade were independently associated with a lower likelihood of de-escalation. Among patients undergoing SLNB, 12.4% had positive SLNs, which is similar to rates of nodal positivity in randomized trials [2, 3], and among patients who underwent completion ALND, the number with additional positive nodes identified was low, further supporting a low underlying burden of nodal disease in this patient population. Notably, a mean of only 3.5 additional nodes were removed among patients undergoing completion ALND, which is fewer than expected. The low mean number of nodes removed likely reflects registry coding, incomplete nodal counts, nodes removed at prior SLNB, or variation in what was coded as ALND, so these counts should be interpreted cautiously.

We also observed patterns that provide insight into how de-escalation strategies are incorporated into clinical practice since both institutional and socioeconomic factors appear to influence the adoption of dual de-escalation. Treatment at an academic center compared to a community center was the strongest predictor of dual de-escalation (OR 2.24, *p* < 0.0001). This potentially reflects greater access to multidisciplinary tumor board input, participation in clinical trials or earlier familiarity with emerging trial data, and a higher institutional comfort level with departing from traditional treatment paradigms. Higher median household income was also associated with dual de-escalation, consistent with previously seen trends where higher socioeconomic status may facilitate the access and flexibility required to navigate complex treatment decisions [12, 13].

Our findings likely reflect a broader pattern of practice evolution that precedes prospective validation rather than deviation from evidence-based care, mirroring the general paradigm shift towards de-escalation in the field of breast cancer surgery over the last 20 years [14]. The adoption of SLNB omission in this cohort began prior to the publications of the SOUND [2], INSEMA [3], and the presentation of mature BOOG 2013-08 [4] follow-up data, suggesting that clinicians implementing these de-escalation strategies were extrapolating from prior data in select populations. We observed higher rates of SLNB omission in our cohort of women 50–69 after the 2016 publication of the Choosing Wisely recommendation for SLNB omission in low risk women ≥ 70 [15]. That recommendation was itself grounded in CALGB 9343, in which axillary surgery was left to surgeon discretion and roughly two-thirds of enrolled patients ultimately received neither axillary dissection nor radiation [16]. At long-term follow-up, overall survival in this cohort matched or exceeded that of an age-matched population without breast cancer, and breast cancer contributed minimally to mortality. However, CALGB 9343 was restricted to women ≥ 70 with small, hormone receptor-positive tumors, a population whose favorable biology and shorter life expectancy together yield a low absolute risk of recurrence. Younger women have a longer period over which locoregional and distant recurrences may emerge, and the trial’s findings should not necessarily be applied to our cohort. Although, the subsequent SOUND and INSEMA trials, which demonstrated non-inferiority of SLNB omission in clinically node-negative patients, have since validated this earlier shift in practice.

APBI use approximately doubled (6.3% to 12.3%) following the 2016 ASTRO guideline update, which expanded recommendations for APBI beyond clinical trial settings [17]. Continued increases in APBI utilization were also seen after the publications of NSABP B-39/RTOG 0413 [18] and Florence [19] trials. Taken together, these findings suggest that individual de-escalation strategies became integrated into routine clinical practice prior to their combined application gaining traction. Notably, performance of SLNB was a strong predictor of APBI (OR 1.89, *p* < 0.001), suggesting that pathologic nodal staging is a strong preference for consideration of APBI. This pattern is consistent with the clinical paradigm in which APBI is used as a targeted treatment, rather than a comprehensive treatment field to include the low axilla, and reserved in patients with pathologically confirmed node-negative disease.

Contemporary data suggest that this pattern continues to evolve. Recent reports demonstrate substantially higher rates of dual de-escalation in select patients, with Memorial Sloan Kettering Cancer Center reporting a rate of 56% of patients meeting criteria for and undertaking SLNB omission also receiving APBI [20]. This marked increase from the rates observed in our study reflect growing comfort with treatment de-escalation in the absence of prospective data validating the combined approach. Importantly, our study period concluded in 2022, prior to the publication of SOUND (2023) and INSEMA (2025) and prior to the initial presentation of BOOG 2013-08 outcomes. The patterns described here represent a pre-trial baseline of dual de-escalation adoption rather than a snapshot of contemporary, evidence-informed practice. Our analysis establishes the reference point against which any acceleration in dual de-escalation following the implementation of trial data can be measured, and characterization of post-publication practice represents an important next step.

An important distinction that emerged from our analysis is the difference between structured de-escalation, and the omission of therapy altogether. While APBI represents a clinician-directed strategy to minimize toxicity while maintaining oncologic intent, radiation omission may be driven by a different set of variables, including patient preference [21] and barriers to care [22]. We observed a strong association between omission of SLNB and receipt of no radiation, suggesting that real-world treatment decisions reflect patient-centered decision-making based on factors such as patient preference, access and barriers to care, or other unmeasured factors in the NCDB. The high rate of nonradiation in patients with SLNB omission, suggests that these unmeasured barriers prevent adherence to standard multi-week radiation regimens. There is likely meaningful overlap between patients who would benefit from omission of additional surgery (SLNB) and those unable to complete a full course of radiation. The potential to shorten courses of radiation therapy allow for vulnerable patients to receive the benefit of adjuvant treatment without forgoing therapy entirely.

The potential combination of these de-escalation strategies raises important considerations for multidisciplinary care. Omission of SLNB eliminates pathological nodal staging, which may influence decisions about systemic therapy. In parallel, substituting APBI for WBI reduces incidental radiation to the low axilla, which may otherwise contribute to treatment of subclinical nodal disease [23]. Together, these strategies may introduce a staging and treatment gap. Because these combined strategies remain unstudied in this population, it may also carry unanticipated implications for both locoregional and distant recurrence. This uncertainty may help explain the reluctance to pair SLNB omission with APBI observed in our cohort. In the setting of absent pathologic confirmation of nodal status, clinicians may have been unwilling to further narrow the radiation field given the risk of undertreating occult nodal disease that would otherwise be addressed by identification on pathologic staging or comprehensive whole-breast field. It is worth noting that the degree to which WBI treats occult axillary disease is variable, and dependent on factors such as beam angle, field borders, and technique, rather than being standardized and varies per patient. Relying on WBI as a safeguard against nodal disease is potentially depending on an imprecise and inconsistent approach unless this is taken into account. This variability may not affect tumor control but can affect comorbidities such as lymphedema [24]. These uncertainties highlight the importance of multidisciplinary coordination and support the development of validated decision aids to guide individualized treatment decisions.

This study has several limitations inherent to its retrospective, registry-based design. The NCDB is subject to selection bias and unmeasured confounding. Although it provides broad national representation, it does not capture detailed clinical decision-making factors, adherence to hormone therapy, or patient preferences, which cannot be fully accounted for despite multivariable adjustment. Additionally, oncologic outcomes beyond overall survival are not available, limiting our ability to assess the short-term safety of these approaches. The NCDB data available at the time of analysis extended only through 2022, preceding availability of data from the SOUND, INSEMA, and BOOG 2013-08 trials. Also, the NCDB does not capture preoperative axillary imaging, which was required to confirm a clinically negative axilla in these trials, which if routinely employed may have decreased the rate of SLNB omission in situations when questionable or positive nodes were discovered. Additionally, year of diagnosis was not included as a model covariate and temporal trends were instead characterized descriptively (Fig. 1). Given the substantial practice evolution over 2012–2022, model associations should be interpreted as average effects across this period rather than time-fixed effects. Ultrahypofractionated WBI was excluded so we could not assess whether shorter WBI schedules altered balance between APBI, WBI, or no radiation. Our models also did not account for clustering of patients within individual facilities. While institution type was included and was a strong predictor across all three outcomes, this only adjusts for average differences between facility categories and not within facility correlation, so standard errors may be underestimated. Updated analysis incorporating subsequent diagnosis years will be needed to characterize dual de-escalation adoption in the post-trial era. Despite these limitations, this study provides important insight into real-world practice patterns during a period of rapid evolution in breast cancer care.

## Conclusion

National practice patterns demonstrate a steady shift toward surgical and radiation de-escalation in early-stage HR+/HER2- breast cancer. Dual de-escalation remained rare and stable after 2018 (0.23–0.26%) reflecting caution among physicians to combine two separate de-escalation modalities. These findings suggest that a small subset of clinicians are extending established de-escalation strategies to combined approaches in selected patients, establishing a cautious pre-trial baseline of early adoption. Prospective studies are needed to define the oncologic safety, optimal patient selection, and quality-of-life outcomes associated with combined de-escalation strategies. Until such data are available, careful multidisciplinary evaluation and shared decision making remain essential.

## Data Availability

The datasets generated during and/or analyzed during the current study are not publicly available due to being protected by the NCDB and are available upon request through the NCDB PUF.
